# Determination of total phenolic content and antioxidant activity of *Commiphora mollis* (Oliv.) Engl. resin

**DOI:** 10.1186/s13065-022-00841-x

**Published:** 2022-06-25

**Authors:** Guyo Jilo Molole, Abera Gure, Negera Abdissa

**Affiliations:** Department of Chemistry, College of Natural Science, Jimma University, P.O. Box 378, Jimma, Ethiopia

**Keywords:** *Commiphora mollis* resin, Folin–Ciocalteu, Total phenolic content, Antioxidant activity, DPPH radical scavenging activity, Reaction kinetics

## Abstract

In this study, total phenolic contents (TPC) and antioxidant activity of *Commiphora mollis* (Oliv.) Engl. (Burseraceae) resin were investigated. The resin was extracted using petroleum ether, chloroform, and methanol to give 27.46 ± 0.48, 46.56 ± 0.42, and 53.00 ± 1.39% extractable solids, respectively. The Folin–Ciocalteu (F–C) redox assay was optimized considering relevant parameters such as reaction time, maximum wavelength, and sample dilution effect before the determination of TPC. The concentration of antioxidants necessary to decrease by 50% the initial concentration of DPPH (EC_50_) was determined at 60 min. The reaction kinetics was analyzed using the pseudo-first-order kinetics model. For the F–C assay, the optimum conditions for the maximum absorbance and analysis time were 760 nm and 30 min, respectively. Under these conditions, the method exhibited good sensitivity and linear instrumental responses over wide ranges of concentrations. The highest TPC;168.27 ± 3.44, 137.43 ± 1.32, and 136.16 ± 0.42 mgGAE/g were recorded in the diluted samples (500 µg/mL) of methanol, chloroform, and petroleum ether extracts, respectively. By using different concentrations of the test sample, exhaustive reduction of phenolics and/or antioxidant substrates was achieved. Regarding the DPPH radical scavenging capacity, the EC_50_ values for methanol, chloroform, and petroleum ether extracts were 295.03 ± 3.55, 342.75 ± 9.72, and 353.69 ± 7.30 µg/mL, respectively. The standard (l-ascorbic acid), however, exhibited much lower EC_50_ value (44.72 ± 0.48 µg/mL). The methanol extracts showed kinetic behavior (k_2_ values,115.08 to 53.28 M^−1^ s^−1^; steady-state time, < 29 min) closer to that of l-ascorbic acid (k_2_ values, 190 to 109 M^−1^ s^−1^; steady-state time, < 16 min), than other two extracts (k_2_ values,14 to 28 M^−1^ s^−1^; steady-state time, 63 to 130 min). For all tested samples, the rate of the DPPH radical scavenging increases with concentration from 50 to 250 µg/mL. The current study demonstrated that the polar solvent (methanol) extract has a better F–C reducing capacity and DPPH radical scavenging activity than the nonpolar solvents extracts. This could be due to phenolics and other oxidation substrates extracted by methanol from the *C. mollis* resin. For a better understanding of the antioxidant constituents of the resin, a further study including isolation of its compounds is recommended.

## Introduction

The free radical reaction is one of the major causes of problems, particularly in the health and food industries. It causes many dreadful diseases including cancer [1] and oxidative rancidity of foods [2]. Synthetic and natural antioxidants are used routinely in the medicine and food industries to minimize oxidative damages [3–5]. However, studies have indicated that synthetic antioxidants are usually associated with adverse effects and potential toxicities [6]. Therefore, natural antioxidant alternatives derived from plants are recommended.

A variety of plant materials are natural sources of antioxidants due to the phytochemicals such as alkaloids, flavonoids, phenolics, and terpenoids they contain [1]. Medicinal plants mainly with documented and popular traditional use in folk medicines for the treatment of oxidative and other related diseases could be a good source for novel and effective antioxidants. Most plants from the genus *Commiphora* (family Burseraceae) produce aromatic resins (myrrh) which have widely been used by the traditional healers for the treatment of different ailments [7]. *Commiphora mollis* (Oliv.) Engl. is among these medicinal plants whose resin has traditionally been used to treat skin inflammation, gastrointestinal disorder, and wounds; particularly for domestic animals in the Southeastern part of Ethiopia. Several studies have indicated that the crude extracts and isolated metabolites from the species of the genus have demonstrated different pharmacological effects including antioxidants [8–13], anti-inflammatory [8] anti-proliferative [11], cytotoxic [9, 14], and anti-hyperglycemic [10] activities. It has also been indicated that sesquiterpenoids, diterpenes, triterpenes, ferulates, and sterols are responsible for antioxidant activities; lignans for the cytotoxicity; and steroids for antiproliferative anti-inflammatory, hypolipidemic, and antidiabetic activities. However, the biological activities and phytochemical information pertaining to *C. mollis* are only limited to the isolation of flavonoids [15] and curcumin [16] from its heartwood and leaf extract, respectively.

Several in vitro techniques have been used for preliminary testing of the antioxidant capacities of plants. Different factors such as extraction solvents type and assay mechanisms for testing antioxidant activities could affect the reliability of results. Both polar and nonpolar solvents were used to prepare crude extracts of resins from the bark of various *Commiphora* trees [8, 11, 12]. Different assay mechanisms including hydrogen atom transfer (HAT), electron transfer (ET), reducing power, and metal chelation [17, 18] can be used for the antioxidant activity testing. Although the assay conditions may differ, correlation analysis of the data might be used as a validation tool.

The 2,2-Diphenyl-1-picrylhydrazyl (DPPH) free radical scavenging and the Folin–Ciocalteu (F–C) redox methods are often used in the analysis of the antioxidant potential and total phenolic contents of medicinal plants, respectively. These methods are simple, rapid, and produce precise results [18]. The DPPH assay is based on ET and/or HAT from the antioxidants to the DPPH free radical. According to the principle, the degree of decolorization of the DPPH solution caused by the neutralization of a comparable number of radicals is measured in terms of absorbance drop, at 517 nm. The EC_50_ value which is the concentration of the antioxidant required to lower the initial DPPH concentration by 50%, is frequently used to describe the antioxidant activity [19, 20]. Indeed, it is important to note that the free radical species that cause oxidative damage in living tissues and/or foods have a very short lifespan. This means that the number of reactive groups per sample is less important than the rate at which each active group may quench the radicals. The only approach to see this feature of a substance is to study its reaction kinetics.

The rate constant is a very important kinetic parameter that shows both the antioxidants concentration and speed of the reaction. The DPPH radical appears to exhibit mainly biphasic interactions with many antioxidants found in plants [20]. Plant extracts that can contain phytochemicals with varying activities can also exhibit mixed reaction mechanisms. In this case, using the pseudo-first-order kinetics model and extracting the sample with different polarity solvents may be beneficial.

The F–C assay is a well-known method for the determination of TPC [21]. This assay is significant in measuring total antioxidant capacity since high phenolic content has been linked to high antioxidant capacity [18]. The original approach has been extensively modified, and several protocols and documented procedures with varying degrees of conditions are available. The F–C assay involves the reduction of the F–C reagent with phenolic compounds in an alkaline medium. The reaction is accompanied by the formation of a blue-colored complex that has a maximum absorbance at 765 nm. The absorbance is directly proportional to the TPC which is reported as Gallic acid equivalent. To obtain reliable data, various conditions such as maximum absorption wavelength (λ_max_), reaction time for color development, and volume ratio of alkali and F–C reagent are required to be studied [22, 23].

The literature survey showed that resins of *Commiphora* plants exhibit antioxidant activities. However, this information is not available for *C. mollis* resin. Therefore, the objective of this study was to determine the antioxidant activity and TPC of *C. mollis* resin crude extracts. TPC and DPPH radical scavenging activity were evaluated by ultraviolet-visible spectrophotometry after linearity of responses at the maximum absorption wavelengths of the corresponding reactions were established. Kinetics analysis was performed to determine the optimum incubation time and rate of radical scavenging effects for the F–C and DPPH assays, respectively. The study also provides information on the antioxidant activity of *C. mollis* resin extracts as evaluated by the EC_50_ (determined at a specific time) and reaction kinetics and the effect of extraction solvents.

## Materials and methods

### Chemicals

All chemicals and reagents used were of reagent grades. Solvents such as petroleum ether, chloroform, and methanol were obtained from Loba Chemie Pvt, Ltd (Mumbai, India). The 2,2-diphenyl-1-picrylhydrazyl (DPPH, 85%) and Folin-Ciocalteu reagent (2 N) were supplied by Alpha Chemika (Mumbai, India) and Sisco Research Laboratories Pvt. Ltd. (Navi Mumbai, Maharashtra, India), respectively. Sodium carbonate anhydrous (99.5%) was purchased from Blulux Laboratory Pvt (Faridabad, Haryana, India). l-Ascorbic acid (99%) and gallic acid (99%) were purchased from Nice Chemicals Pvt (Ernakulum, Kerala, India).

### Instrument

Double-beam UV/Vis spectrophotometer (SPECORD 200 PLUS, Analytik Jena, Germany) was used for quantitative analysis.

### Sample collection

The resin of *C. mollis* was collected from Gorile, Das district, Borana Zone, Oromia Regional State, Ethiopia, in February 2020. The collected samples were shade dried and double-sealed with a plastic bag and transported to the Department of Chemistry Laboratory, Jimma University, for further treatment and analysis. The plant material was identified by Botanist (Dr. Dereje Denu) and the voucher specimen, CH8-JUH has been stored in Jimma University Herbarium.

### Preparation of resin extracts

The pulverized resin (15 g) was transferred to three amber bottles. Then, 200 mL of methanol, chloroform, and petroleum ether were separately added. The content was shaken for 15 min on the shaker and then stored in the dark at room temperature. After 24 h, the infusions were filtered with Whatman No. 1 filter paper. The obtained residue was re-extracted with an equal volume of the same solvents. Finally, the filtrates were combined and evaporated to one-quarter of their volume using a rotary evaporator at 40 °C. The obtained extracts were allowed to dry at room temperature and the extraction yield was expressed as a percent of dry extract per gram of the dry resin [12].

### Total phenolic contents

The F–C method reported by Ainsworth and Gillespie [27] and Adusei et al. [24] was used with slight modification. During the experiments, the reagents, and sample solutions were prepared as follows: The F–C reagent was diluted to 1:10 with distilled water just before the experiment. Sodium carbonate (7.5% w/v) was also prepared in distilled water. Stock solutions of each dry extract of *C. mollis* resin (1000 µg/mL) and the standard compound, Gallic acid, (500 µg/mL) were prepared in methanol (95% vol/vol).

#### Determination of maximum absorption wavelength and time

Different solutions of Gallic acid (50 and 100 µg/mL) and *C. mollis* resin extracts (200 and 400 µg/mL) were prepared from the stock solutions. Following the general procedure, 0.5 mL of each of these solutions or blank (methanol, 95% v/v) was transferred to screw-capped tubes and mixed with 2 mL F–C reagent using a vortex mixer for a few seconds. After 3 min but before 8 min, 4 mL of Na_2_CO_3_ was added and mixed well. The sample was then transferred to a quartz cuvette and scanned in the wavelength range of 400 to 900 nm from 5 to 60 min at 5 min intervals. The obtained spectra and absorbance data were used to determine the λ_max_ and the optimum reaction time [25].

#### Determination of TPC

For the determination of TPC of *C. mollis* resin extracts, three independent samples of each extract (500 and 1000 µg/mL) were used. Gallic acid calibration solutions of 0, 25, 50, 75, 100, 125, and 150 µg/mL concentrations were prepared in duplicates. Then, the reaction mixtures were prepared following the procedure earlier stated and the absorbance was recorded at previously determined λ_max_ (760 nm) and incubation time (30 min). All measurements were performed in triplicates. The average absorbance of the two-calibration series was used for the construction of the calibration curve. The coefficient of determinations (R^2^) of the calibration curve was used to evaluate the linearity of the curve. Limit of detection (LOD) and limit of quantitation (LOQ) were calculated as 3.3σ/s and 10σ/_S_, respectively, where σ is the standard deviation of the response, and S is the slope of the calibration curve [26]. The concentration of Gallic acid in each extract was calculated from the regression equation using their absorbance. Finally, these results were converted to the TPC as milligrams of Gallic acid equivalent per gram of dry extract (mg GAE/g) using Eq. (1) [27].

1 $${\text{C}} = {\text{C}}_{1} \times \frac{{\text{V}}}{{\text{m}}}$$

where C is TPC in mg/g, in GAE (Gallic acid equivalent), C_1_ is the concentration of Gallic acid established from the calibration curve in mg/mL, V is the volume of the extract in mL, and m is the weight of the dry plant extract in g.

### DPPH radical scavenging activity

The DPPH radical scavenging activity of the extracts was evaluated using the procedure described by Brand-Williams with some modifications [19]. A solution of 101.44 µM DPPH was prepared by dissolving 0.01 g of DPPH in 25 mL methanol. Dilutions were made to obtain 10, 20, 30, 40, 50, and 60 µM DPPH solutions. Each of these solutions was transferred to a cuvette and scanned over a wavelength range of 400–600 nm against the blank (methanol). The spectra and absorbance data were used to find the λ_max_ and to establish the linear relationship between absorbance and concentration of the standards.

#### ***Determination of EC***_***50***_

The stock solutions of the *C. mollis* resin extracts (1000 µg/mL) and l-ascorbic acid (800 µg/mL) were separately prepared in methanol. Then, a series of solutions of 400, 350, 300, 250, 200, 150, 100, and 50 µg/mL were separately prepared in triplicates from each stock solution. Afterward, 1 mL of each of these solutions was mixed with 2 mL of 40 µg/mL DPPH and shaken vigorously. The control sample was also prepared by mixing 1 mL of methanol with 2 mL of 40 µg/mL DPPH. The mixture was then incubated in dark for 60 min at room temperature. Eventually, the absorbance of the mixture was measured at λ_max_ (517 nm). The exact concentration of DPPH in the reaction medium was calculated using the calibration curve equation and the remaining percent of DPPH was calculated by Eq. (2):

2 $$\% \;{\text{of}}\;{\text{remaining}}\;{\text{DPPH}} = \frac{{{\text{A}}_{{\text{s}}} }}{{{\text{A}}_{0} }} \times 100$$

where A_0_ is the absorbance of the DPPH solution without antioxidants and A_s_ is the absorbance of the sample [28].

The obtained % of DPPH was plotted against the concentration of the samples to calculate EC_50_ values.

#### Kinetics analysis

Kinetics of the DPPH radical scavenging activity of *C. mollis* resin extracts and standard was studied by the previously reported method [20] with modification. Accordingly, an aliquot (1 mL) of a solution containing different concentrations of the sample or standard (250, 100, and 50 µg/mL) was mixed with 2 mL of 101.44 µM DPPH solution. Similarly, a control sample (DPPH) was prepared by mixing 1 mL of methanol with 2 mL of 101.44 µM DPPH. Then, the decrease in absorbance was measured at 517 nm, 2 min after the addition of DPPH and at regular intervals of 2 min for the first 15 min and, subsequently, every 5 min until the reaction approaches its steady state.

The kinetics of the DPPH radical scavenging reaction depends on the concentrations of both reactants. So, the rate of reaction was defined as

3 $$- \frac{{{\text{d}}\left[ {{\text{DPPH}}} \right]}}{{{\text{dt}}}} = {\text{k}}_{{1}} \left[ {{\text{DPPH}}} \right] = {\text{k}}_{2} \left[ {{\text{DPPH}}} \right]\left[ {{\text{Antioxidant}}} \right]$$

where k_1_ and k_2_ represent the pseudo-first-order and second-order rate constants, respectively.

In all cases, the initial concentration of antioxidants in the medium was lower than that of DPPH. This condition was fulfilled when some DPPH concentrations remained in the medium at the end of the reaction. Thus, the reaction is pseudo-first-order to antioxidant groups in the tested samples which completely disappeared from the reaction medium according to Eq. (4).

4 $${\text{A}} = {\text{A}}_{0} {\text{e}}^{{ - {\text{K}}_{1} {\text{t}}}}$$

where A is the scavenged concentration of DPPH at any time t, A_0_ is the span of the reaction (the total amount of DPPH scavenged at a steady-state), k_1_ is the apparent first-order rate constant, which describes the velocity of the disappearance of antioxidants from the reaction medium.

Finally, k_2_ was determined from the initial concentration of DPPH according to Eq. (4). In addition, half-life and steady-state time of the reaction was used to compare the reactivity of the tested samples. All the kinetic parameters were determined by fitting the obtained data using the Levenberg–Marquardt method [29] implemented in Graph pad prism version 8.0.2 software.

### Statistical analysis

All measurements were conducted in triplicates and the final results were reported as mean with the standard deviation (mean ± SD). Statistical analysis was performed using Graph Pad Prism version 8.0.2. Analysis of variance (ANOVA) with Tukey’s multiple comparison test (P < 0.05) was performed to compare the mean values of the results.

## Results and discussion

### Extraction yield

The extraction step is crucial to isolate active compounds from plant materials and reduce interferents [30]. Methanol, chloroform, and petroleum ether extracts of *C. mollis* resin were prepared under the same conditions. The results showed that methanol yielded the highest extractable solids than other solvents. The yields of extractable solids were 53.00 ± 1.39, 46.56 ± 0.42, and 27.46 ± 0.48% for methanol, chloroform, and petroleum ether, respectively, showing the dependence on the polarity of extraction solvents. Since plants can contain polar or non-polar compounds, the polarity of the extraction solvents plays a critical role in separation purposes [30].

### Total phenolic contents

**Fig. 1:**
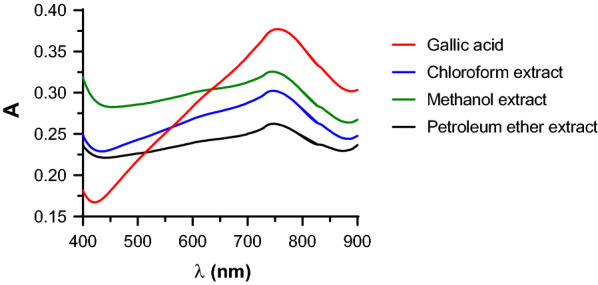
Absorption spectra (400 to 900 nm) of the product of the reaction of F-C reagent with Gallic acid (50 µg/mL) and *C. mollis* resin extracts (200 µg/mL)

A group of compounds with similar chemical structures may show the same chemical interactions with a specific reagent during the reaction. The F–C assay is a reaction based on an ET from phenolic compounds and other oxidation substrates to the F–C reagent, phosphomolybdic/phosphotungstic acid complexes (H_3_PW_12_O_40_/H_3_PMo_12_O_40_), resulting in blue complexes (possibly (PMoW_11_O_40_)^4–^) which induce λ_max_ at about 765 nm [23]. Deviation from the λ_max_ can result from oxyreduction responses of the F–C reagent [25] and the complex structural differences of polyphenols (antioxidants) occurring in plants [31]. This deviation could be capable of compromising the experimental responses by underestimating the quantitative data. Therefore, the absorption spectra of the *C. mollis* resin extracts was compared with that of the reference compound, Gallic acid. All the crude extracts show an approximately similar absorption spectrum as the reference compound (Fig. 1).

As can be seen from Fig. 1, the λ_max_ lies between 700 and 800 nm. Furthermore, Table 1, shows an insignificant change (coefficient of variation, CV ≤ 0.65%) in absorbance values in a range of 740 nm to 770 nm. In line with the standard method [22] and other preceding studies [25, 31, 32], a wavelength of 760 nm was chosen.

**Table 1:** The absorbance of solutions of Gallic acid and *C. mollis* extracts after 30 min of reaction with F–C reagent in a wavelength range of 740–770 nm

Sample	Conc. (µg/mL)	Absorbance at the wavelength (nm) of						
		740	745	750	755	760	765	770
Gallic acid	50	0.374	0.376	0.377	0.377	0.377	0.376	0.374
	100	0.874	0.876	0.878	0.879	0.880	0.880	0.880
Chloroform extracts	200	0.302	0.302	0.302	0.302	0.300	0.299	0.297
	400	0.460	0.461	0.461	0.461	0.461	0.460	0.459
Methanol extracts	200	0.325	0.326	0.325	0.324	0.323	0.322	0.319
	400	0.482	0.483	0.483	0.483	0.483	0.482	0.481
Petroleum ether extracts	200	0.262	0.262	0.262	0.262	0.261	0.260	0.259
	400	0.415	0.416	0.416	0.416	0.415	0.415	0.414

**Fig. 2:**
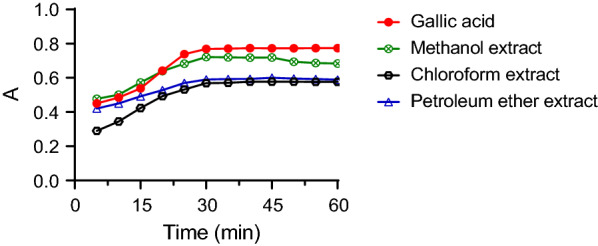
Change in absorbance with a reaction time of F-C reagent with Gallic acid and *C. mollis* resin extracts

The optimal time for color development was determined using the selected wavelength. According to the reaction kinetics, the absorbance increased from 5 to 30 min, for all samples tested, then remained steady for a while before progressively dropping (Fig. 2). Despite this, for Gallic acid, the absorbance was remarkably constant throughout the experiments from 30 to 60 min (CV ≤ 0.25%). However, for *C. mollis* resin extracts, the optimal responses were observed between 30 and 45 min (CV ≤ 0.65%).

TPC determination at steady-state improves the precision of analytical results. Conditions like high temperature and alkali levels, accelerate color development and fading [22, 23]. Thus, studying reaction kinetics can provide useful information. Results of the current study demonstrated that 30 min of incubation time is required before measurement of the absorbance. The obtained findings were in agreement with previous reports on optimization of the F–C method for the determination of TPC from plant extracts [25, 31].

**Fig. 3:**
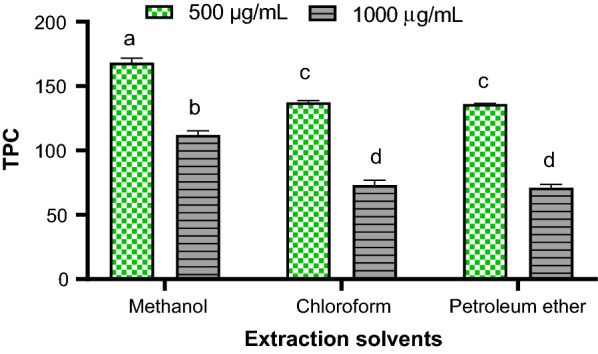
TPC of *C. mollis* resin extracted using the different solvents. Values followed by different letters are significantly different (ANOVA with Tukey’s test, n (instrumental × sample replicate) = 9, p < 0.05)

To determine the TPC in the resin extracts, under the specified conditions, a calibration curve was constructed using Gallic acid calibration standards (0 to 150 µg/mL). The coefficient of determination (R^2^) of the resulting calibration curve (y = 0.0082x + 0.0108) was 0.9997, suggesting excellent linearity in the studied range of concentrations. The LOD and LOQ of the method were 2.41 and 7.29 µg/mL, respectively. The TPC of *C. mollis* resin extracted by different solvents was calculated using the calibration curve equation and reported as mg GAE/g dry mass of the extract. The results for test samples at 500 and 1000 µg/mL concentration is shown in Fig. 3.

Independent of the extract types, 500 µg/mL provided the higher amount of TPC, i.e. the TPC was 168.27 ± 3.44, 137.43 ± 1.32, and 136.16 ± 0.42 mg GAE/g for methanol, chloroform, and petroleum ether extracts, respectively. At 1000 µg/mL, 112.44 ± 3.33, 73.91 ± 3.87, and, 71.94 ± 2.51 mg GAE/g were obtained for methanol, chloroform, and petroleum ether extracts, respectively. Apart from the position of λ_max_ and optimal reaction time for color development, an excess of F–C reagent is recommended for the exhaustive reduction of phenolic antioxidants in the sample [22]. The obtained lower TPC in more concentrated samples could be due to an insufficient amount of the F–C reagent in the reaction medium. By varying concentrations of the extracts, enhanced responses could be observed.

TPC is one of the important parameters of total antioxidant capacity (TAC) and is widely used for the evaluation of the antioxidant properties of plants [21]. Since plants contain a diversity of phenolics and antioxidant substrates with different structures, molecular sizes, and polarities, the nature of extraction solvents can greatly affect their results [33]. In the present study, polar solvent (methanol) showed the highest TPC than non-polar solvents (petroleum ether and chloroform). Besides, the TPC of petroleum ether and chloroform did not differ significantly (P > 0.05). In previous studies, methanol was also chosen for the extraction of phenolic contents from different plant samples [34–36]. This indicates better solubility of these compounds in polar solvents. Generally, the findings demonstrated that *C. mollis* resin contains a high amount of TPC. Such high TPC in plants has a correlation with different pharmacological effects [21].

### DPPH radical scavenging activity

**Fig. 4:**
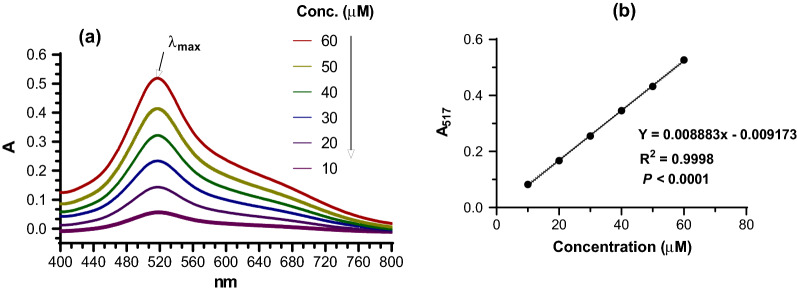
(**a**) Absorption spectra (400 to 800 nm) for solutions containing 10, 20, 30, 40, 50, and 60 μM DPPH and (**b**) linear relationship between concentration and absorbance measured at 517 nm

The ability of antioxidants to reduce the DPPH radical concentration in the reaction medium is assessed spectrophotometrically by monitoring the drop in absorbance at a characteristic wavelength [17]. As a result, the maximum absorption wavelength, as well as the linearity of the instrumental response to DPPH concentration, had to be determined. According to the findings, the maximum absorption was found at 517 nm (Fig. 4a). In addition, a linear relationship was established between DPPH radical concentration and measured absorbance at 517 nm (Fig. 4b).

**Fig. 5:**
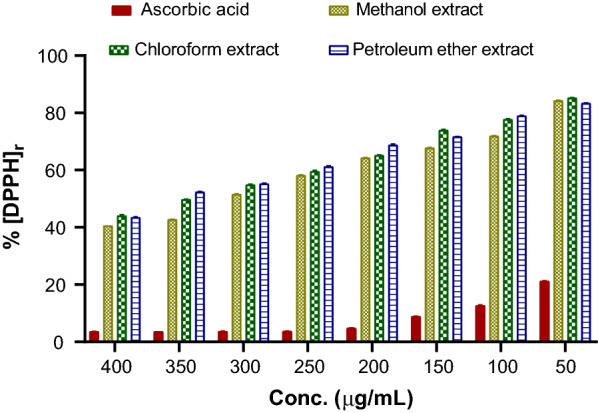
DPPH radical scavenging effects of *C. mollis* resin extracts and ascorbic acid. Results were expressed as mean ± standard deviation of three means (n = 3)

DPPH is one of the free radicals widely used for testing the preliminary antioxidant activity of plant extracts. The decrease in absorbance of DPPH solution is proportional to the antioxidant concentration [19]. In this study, a DPPH radical scavenging capacity increased with increasing concentrations of extracts. Methanol extract of *C. mollis* resin, displayed the highest DPPH radical scavenging activity at most of the tested concentrations levels (Fig. 5). When 400 µg/mL extract of each solvent: methanol, chloroform, and petroleum ether were reacted with DPPH 59.43 ± 0.37%, 55.95 ± 1.41%, and 56.52 ± 0.90% of the initial concentration of DPPH were scavenged, respectively. An equal concentration of the ascorbic acid scavenged 96.39 ± 0.03% of the initial concentration of DPPH.

The effective concentration that causes a decrease in the initial DPPH concentration by 50% is defined as EC_50._ The substance with lower EC_50_ is considered to exhibit significant antioxidant activity [19]. The EC_50_ values of *C. mollis* resin extracts were 295.03 ± 3.55, 342.75 ± 9.72, and 353.69 ± 7.30 µg/mL for methanol, chloroform, and petroleum ether extracts, respectively. These results revealed that methanol extract has the highest antioxidant activity than chloroform and petroleum ether extracts, but has lower antioxidant activity compared to the ascorbic acid (EC_50_ = 44.72 ± 0.48 µg/mL). Besides, statistical analysis showed that EC_50_ values of chloroform and petroleum ether extracts were not significantly different (P > 0.05). These outcomes demonstrated that polar solvent (methanol) is more effective for the extraction of antioxidants than non-polar solvents (chloroform and petroleum ether). Moreover, a similar pattern was observed for TPC**.** That means methanol extract contained the highest amount of TPC compared to the other two solvents. Previous studies also demonstrated a positive relationship between the DPPH and F–C assays [34–37].

Compared to resins of other *Commiphora* species, methanol extract of *C. mollis* exhibited greater DPPH inhibition effects. For instances, *C. myrrh* resin extracts obtained by various solvents demonstrated different DPPH inhibition effects (methanol extract, EC_50_ = 320 µg/mL; ethyl acetate extract, EC_50_ = 930 µg/mL, essential oil, EC_50_ = 11,330 µg/mL) [12]. Hexane extract of *C. erythrea* resin exhibited EC_50_ > 3000 µg/mL [8].

### Kinetics analysis

**Fig. 6:**
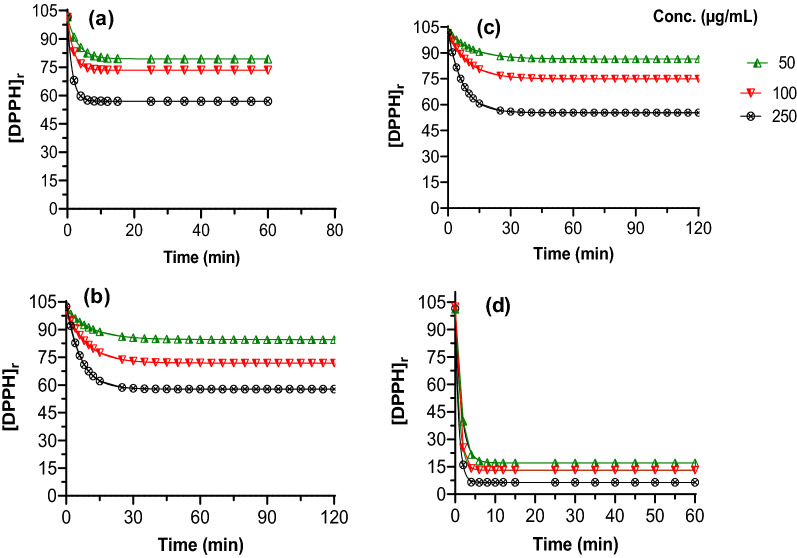
Kinetics analysis of DPPH radical scavenging reaction of (**a**) methanol, (**b**) chloroform, and (**c**) petroleum ether extracts of *C. mollis* resin and (**d**) l-ascorbic acid. The graphs were plotted as the remaining concentration of DPPH, ([DPPH]_r_) versus time

The kinetics of the DPPH radical scavenging reaction of the standard (l-ascorbic acid) and *C. mollis* resin extracts was studied as a function of time. Throughout the experiment, the absorbance of the control sample was monitored to ensure that the DPPH radical remained stable in the reaction medium. Figure 6 illustrates the DPPH radical scavenging profile for the tested samples. The differences in the manner of loss of DPPH with time can be marked by the difference in the shapes of reaction curves.

The radical (DPPH^⋅^) is reduced via HAT, ET, or a combination of the two mechanisms. The HAT reaction is a direct abstraction of H-atom from the antioxidant, RH by the free radical, DPPH^⋅^ (Eq. 5) [17]. This reaction is governed by the hydrogen bond dissociation enthalpy (BDE) of the compound.

5 $${\text{RH}} + {\text{DPPH}}^{ \cdot } \to {\text{R}}^{ \cdot } + {\text{DPPHH}}$$

In the ET reaction, an electron is transferred from ionic intermediate to DPPH^·^. The following reactions (Eqs. 6–8) show the ET mechanism in an H-bond-accepting solvent (S) [17].

6 $${\text{RH}} + {\text{S}} \to {\text{R}}^{ - } + {\text{SH}}$$

7 $${\text{R}}^{ - } + {\text{SH}} + {\text{DPPH}}^{ \cdot } \to {\text{R}}^{ \cdot } + {\text{DPPH}}^{ - } + {\text{SH}}$$

8 $${\text{DPPH}}^{ - } + {\text{SH}} \to {\text{DPPHH}} + {\text{S}}$$

The ET reaction in polar solvents like methanol, which is often used in the DPPH assay, follows a sequential proton-loss electron transfer (SPLET) steps [17]. The reaction is dependent on the de-protonation ability and ionization potential (IP) of the antioxidants [38].

The kinetics of the reaction also depends on the relative concentrations of DPPH and antioxidants [28]. The reactions followed the pseudo-first-order because the concentration of DPPH was higher than that of the samples. Table 2 summarizes the concentration of unreacted DPPH at the steady-state, reaction half-life and steady-state time, and rate constants. According to the results, the amount of DPPH removed from the medium was proportional to the concentration of the samples. In comparison to l-ascorbic acid the *C. mollis* resin extracts scavenged a smaller quantity of the free radical. At the same concentration, the amount of DPPH that remained at a steady state for *C. mollis* resin extracts was close (CV < 2.25%), but the kinetic variables were different.

**Table 2:** Kinetic data of DPPH radical scavenging activity of ascorbic acid and *C. mollis* extracts at different concentrations

Sample	Concentration (µg/mL)	Half-life, t_1/2_ (min)	Steady-state time(min)	k_2_(M^−1^ s^−1^)	Remaining [DPPH] at a steady-state
Methanol extract	250	0.99	16.53	115.08	56.98
	100	1.31	21.94	87.13	73.4
	50	2.13	28.04	53.28	84.4
Chloroform extract	250	4.57	63.49	24.51	57.77
	100	6.40	94.58	17.94	71.88
	50	7.63	129.58	14.96	84.61
Petroleum ether extract	250	4.85	66.97	23.39	55.30
	100	6.65	96.34	17.04	74.92
	50	7.66	112.93	14.68	86.50
Ascorbic acid	250	0.60	10.02	189.18	6.43
	100	0.70	13.15	162.41	13.06
	50	1.04	15.82	109.80	17.05

The half-life (t_1/2_) indicates the time it takes for antioxidants to reach half of their total radical scavenging activity. This value was less than 3 min for methanol extracts while it ranges from 4 to 8 min for chloroform and petroleum ether extracts. The steady-state reaction time also indicated maximum radical scavenging activity of  the methanol extracts, i.e., the reaction was completed within 16–29 min depending on the concentration of test samples. For the other two extracts, it took more than 1 h to reach the plateau indicating their slow radical scavenging potentials (Table 2).

Compounds with maximum rate constant (k_2_) are considered to be efficient radical scavenging agents [20]. As can be seen in Table 2, the values of k_2_ were the highest for ascorbic acid showing its superior antiradical efficiency. Regarding *C. mollis* resin extracts, methanol extracts have maximum k_2_ values indicating that it is more reactive to the DPPH radical than chloroform and petroleum ether extracts. The kinetic profiles of these two extracts revealed that the reaction is relatively rapid in the initial minutes and slowly declined over a longer time (Fig. 6).

Rate constants refer to the ability of electron-or-hydrogen atom donation of antioxidant groups in the tested samples. Generally, the reactivity and mechanism of radical scavenging depend on the solvent polarity and H-bond strength, concentration, and other structural features of antioxidants such as the presence of oxygen, an aromatic ring, or conjugated double bonds [39, 40]. The ET reaction may be slower than the HAT due to the time required for the reaction to complete and for the solvent to stabilize [38].

Plant extracts could contain a combination of antioxidants with differing reactivity to the DPPH radical. Previous studies have shown that the antioxidant activities of terpenoid groups such as sesquiterpenoids, triterpenoids, furanosesquiterpenoids, and sterols are responsible for the DPPH radical scavenging effect of *Commiphora* trees resins. Demonstration on a kinetic model of DPPH radical scavenging activity of common terpenoids [39], which were also detected in *Commiphora* trees resins [7–9, 12] showed that monoterpenes with conjugated double bonds such as myrcene and γ-terpinene exhibited rapid kinetic behavior due to electron delocalization capacity on their conjugated double bonds (π bond). Terpenes without conjugated double bonds displayed slower DPPH radical scavenging behavior [39].

Phenolic compounds are considered to be extremely important among plant-based antioxidants. Phenolic antioxidants are found in stem and leaves of *C. mollis* [15, 16]. A study on *C. wightii* resin indicated ferulates to be responsible for its DPPH radical scavenging activity [13]. According to Villaño et al. [20], phenolic antioxidants can exert rapid initial depletion of DPPH due to a fast abstraction of H-atom, the followed slow decay was ascribed to the termination step which could be slower as the radical–radical interaction is sterically hindered. Since plant extracts could constitute a mixture of antioxidants with differing reactivity, different kinetic behavior is possible.

## Conclusion

The TPC and antioxidant activity of methanol, chloroform, and petroleum ether extracts of *C. mollis* resin were investigated. Methanol extract showed the highest extraction yield, TPC and DPPH radical scavenging activity. The TPC was determined at 760 nm, after interaction of the extracts with the F–C reagent for 30 to 40 min. Regardless of the type of extracts, the response was maximum for 500 µg/mL sample solution as compared to 1000 µg/mL, underlining the effect of sample to F–C reagent concentration ratio on the TPC. This ensured the exhaustive reduction of phenolic antioxidants in the test samples. Determination of the EC_50_, at 60 min of reactions, and kinetics of the DPPH radical scavenging reactions demonstrated the maximum antioxidant activity of methanol extract. It was also observed that the remaining concentration of the DPPH at a steady-state differed slightly for the three extracts. Nevertheless, the kinetics analysis demonstrated the superior effect of methanol extract as evidenced by the larger k_2_ values which were closer to that of l-ascorbic acid. This implies the importance of kinetic analysis in DPPH assay. Results further revealed the positive correlation between TPC and antioxidant activity. Methanol extract showed the highest TPC and the DPPH radical scavenging activity than other two extracts which showed similar effects. For a better understanding, further in vitro and in vivo studies supported by isolation and structure elucidation of individual component compounds are recommended.

## Data Availability

The datasets generated and/or analyzed during the current study are included in the manuscript. Moreover, the datasets used to generate figures and results are available from the corresponding author on reasonable request.

## Abbreviations

% [DPPH]r: Percent of DPPH concentration remained
ANOVA: Analysis of variance
CV: Coefficient of variation
DPPH: 2,2-Diphenyl-1-picrylhydrazyl
EC_50_: Amount of antioxidant necessary to decrease the initial DPPH concentration by 50%
ET: Electron transfer
F–C: Folin–Ciocalteu
HAT: Hydrogen atom transfer
LOD: Limit of detection
LOQ: Limit of quantitation
mg GAE/g: Milligrams of gallic acid equivalent per gram
SPLET: Sequential proton-loss electron transfer
TAC: Total antioxidant capacity
TPC: Total phenolic content
UV–Vis: Ultraviolet–visible
